# Melatonergic Regulation of Polyethism and Circadian Foraging in *Apis mellifera*

**DOI:** 10.3390/ijms27010035

**Published:** 2025-12-19

**Authors:** Naznin Nahar, Quynh Tranthi, Jadwiga Bembenek, Ahmed A. M. Mohamed, Qiushi Wang, Susumu Hiragaki, Rasha K. Al-Akeel, Hend M. Alharbi, Azza Elgendy, Abdo A. Elfiky, Amr Mohamed, Makio Takeda

**Affiliations:** 1 Graduate School of Agricultural Science, Kobe University, 1-1 Rokkodai, Nada-ku, Kobe 657-8501, Japan; 2 Department of Entomology, Faculty of Agriculture, Bangladesh Agricultural University, Mymensingh 2202, Bangladesh; 3 Department of Zoology, Faculty of Science, King Saud University, Riyadh 11451, Saudi Arabia; 4 Department of Biology, College of Science, Princess Nourah bint Abdulrahman University, Riyadh 11671, Saudi Arabia; 5 Department of Biotechnology, Faculty of Science, Cairo University, Giza 12613, Egypt; 6 Department of Biophysics, Faculty of Science, Cairo University, Giza 12613, Egypt; 7 Department of Entomology, Faculty of Science, Cairo University, Giza 12613, Egypt

**Keywords:** melatonergic signaling, nurse–forager transition, *Apis mellifera*, melatonin receptor, arylalkylamine *N*-acetyltransferase, circadian regulation

## Abstract

Melatonin is a conserved indolamine implicated in circadian and developmental timing, but its role in social-insect task allocation is unclear. Here, we show that melatonergic signaling modulates the nurse → forager transition in the honey bee (*Apis mellifera*). A single hemocoelic dose of melatonin (100 ng) markedly reduced hive retention and advanced the age at first waggle dance by ≈9 days (median 11.8 vs. 20.9 days; common-language effect size = 0.94). Complementary manipulations—pharmacological antagonism with luzindole and RNA interference (RNAi)-mediated knockdown of a candidate melatonin receptor (*AmMTR*/*AmMT2*; transcript reduction ≈65–79% at 24–72 h)—produced reciprocal suppression of foraging, indicating pathway dependence. Transcriptional profiling revealed a forager-age peak in the arylalkylamine *N*-acetyltransferase ortholog *AmNAT2* (≈10-fold increase near day 23), while *AmNAT1* remained unchanged; melatonin treatment was associated with a trend toward increased *Amα-glucosidase* expression. Computational analyses classify AmMTR as a class-A GPCR and identify plausible melatonin-compatible pockets; promoter scans reveal high-confidence circadian motif matches upstream of *AmMTR*. These in silico results are presented as hypothesis-generating. Together, the behavioral, molecular, pharmacological and computational lines of evidence support melatonin as a circadian-informed modulatory signal that helps align neuroendocrine and metabolic states with the timing of extranidal behavior. Confirmation via receptor functional assays and broader colony replication will be important.

## 1. Introduction

Honey bee (*Apis mellifera*) colonies show temporal polyethism: young workers (≈1–12 days) perform intranidal tasks (brood care, wax work, maintenance) and, as they mature (≈14–21 days), shift to extranidal roles like guarding and foraging [1,2]. This transition involves physiological changes—active hypopharyngeal glands (HPGs) in nurses versus HPGs regression and metabolic reorientation toward carbohydrate-fueled flight in foragers [3,4,5,6,7]—and task-linked secretions: nurses’ HPGs produce brood factors (e.g., royalactin) while foragers express enzymes such as α-glucosidase to process nectar into honey [8,9,10,11,12,13]. Brain α-glucosidase reliably marks the nurse → forager shift [14,15]. The trajectory is plastic—workers can accelerate, delay, or reverse roles in response to colony needs—implying integrated regulation by internal physiology and external social/environmental cues [16,17].

The classical juvenile hormone–vitellogenin (JH–Vg) double-repressor model explains much of maturation—high Vg keeps workers in nursing by suppressing JH, while rising JH in foragers suppresses Vg and promotes extranidal behavior [18,19,20,21]—but it does not account for all cases (e.g., allatectomized workers still forage, and JH manipulations do not affect circadian locomotor activity [18,19,22]). Neuromodulators (dopamine, serotonin/5-HT, octopamine/OA) are now recognized as important regulators of maturation and responsiveness [23,24,25,26,27]; OA in particular alters sucrose responsiveness and can induce precocious foraging independently of JH [7], and it also influences circadian-linked per expression without changing the central pacemaker [28]. Recent work implicates brain lipidomic shifts and broad transcriptional reprogramming (e.g., *Broad Complex* dynamics), indicating flexible gene regulatory control rather than single-axis endocrine causation [2,3].

Ontogeny of circadian rhythms is central: nurses are largely arrhythmic (round-the-clock brood care) while foragers show strong diel rhythms [4,29]. This behavioral switch mirrors age/task differences in clock genes (e.g., *period*, *cryptochrome*) and indolamine metabolism enzymes [29,30,31,32,33,34]. Arylalkylamine *N*-acetyltransferase (AANAT), which converts 5-HT to *N*-acetylserotonin (a melatonin precursor), is several-fold higher in foragers, paralleling elevated brain melatonin and suggesting melatonin’s role in circadian behavioral control [35,36,37,38]. In other insects (e.g., *Antheraea pernyi*), AANAT-driven melatonin rhythms regulate neuroendocrine outputs and photoperiodic entrainment, with receptor antagonists like luzindole disrupting these processes [39]; by analogy, higher melatonin in foragers may shape honey bee circadian plasticity [40].

These data motivate the view that the 5-HT → AANAT → melatonin axis functions as a neuroendocrine “gatekeeper” of behavioral maturation: low melatonin in nurses supports arrhythmic physiology and HPGs activity, whereas rising melatonin with age promotes clock gene activation and foraging competence [2,34,38]. Putative receptors (e.g., AmMTR) could interface with OA and JH pathways to tune neural circuits for task allocation. Supporting evidence includes melatonin’s enhancement of antioxidant capacity and cold tolerance (relevant to forager survival) and identification of insect melatonin receptors (e.g., *AccMTNR1A* in *A. cerana*) linking melatonin to stress responses [41,42,43,44,45,46]. Detection of 5-HT/melatonin metabolites in honey raises the possibility of colony-level signaling [37], and melatonin may modulate OA/JH axes to influence task allocation [4,38].

To generate testable receptor hypotheses we built atomistic models of the putative honey bee melatonin receptor (AmMTR) using contemporary deep-learning/GPCR refinement and benchmarked these against human MT2 X-ray structures [47,48]; ligand binding was probed with subsequently assessed through computational docking and interaction profiling to nominate conserved contact residues and insect-specific pocket features for future mutagenesis or biochemical tests [49].

Here we test whether indolamine acetylation and melatonin signaling causally contribute to age-dependent behavioral maturation in *A. mellifera*. Using exogenous melatonin, pharmacological antagonism (luzindole, an MT_2_-preferring melatonin receptor antagonist), RNAi knockdown of the putative receptor (*AmMTR*/*AmMT2*), and natural-colony behavioral assays, we evaluate whether perturbing the melatonin pathway alters (1) probability and timing of hive exit and first waggle dance (foraging onset), (2) brain expression of a forager marker (*Amα-glucosidase*) and dynamics of *AmNAT1*/*AmNAT2*, and (3) receptor transcript abundance/function. We pair these experiments with in silico structural modeling (class-A GPCR classification, candidate ligand pockets) and promoter scans showing circadian motif enrichment upstream of *AmMTR*, thereby testing causality, receptor dependence, and circadian integration of the 5-HT → AANAT → melatonin axis in behavioral maturation. Demonstrating that melatonin signaling can both advance foraging and be necessary for normal foraging propensity—while linking receptor structure and clock-regulated transcription—would integrate indolamine pathways into endocrine and social-cue frameworks for division of labor.

## 2. Results

### 2.1. Behavioral Changes in Nursing Bees After Injections of Melatonin

**Figure 1:**
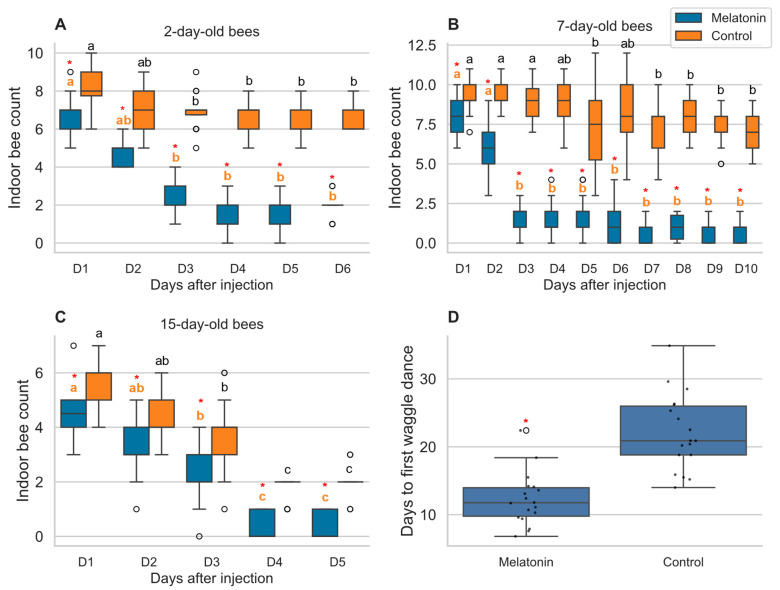
The effect of melatonin on honey bee hive behavior and foraging ontogeny. (**A**–**C**) Indoor hive counts of (**A**) 2-day-old (6 days post-injection, *n* = 24), (**B**) 7-day-old (10 days post-injection, *n* = 30), and (**C**) 15-day-old bees (5 days post-injection, *n* = 18) after a single injection of 100 ng melatonin or vehicle control. (**D**) Days to first performance of the waggle dance after melatonin treatment. Data are mean ± SD. Different lowercase letters denote significant differences over time within a treatment group (Friedman test with Nemenyi post hoc, *p* < 0.05; orange for melatonin, black for control). Asterisks (*) indicate a significant difference from the control group at a given time point (**A**–**C**) or overall (**D**) (Mann–Whitney *U*-test, *p* < 0.05). Open circles represent statistical outliers.

To investigate the effects of melatonin on honey bee hive behavior, we monitored indoor bee counts following a single injection of 100 ng melatonin or vehicle control across three different age cohorts. Melatonin treatment resulted in dramatic and consistent reductions in indoor hive activity compared to controls across all age groups tested (Figure 1A–C).

In 2-day-old bees monitored for 6 days post-injection, melatonin treatment caused a progressive decline in indoor bee counts over time (Friedman test: χ^2^ = 101.82, *p* < 0.001; Figure 1A). Indoor counts in melatonin-treated bees decreased significantly from Day 1 (median = 7.0 bees) to Day 3 (median = 2.0 bees, *p* < 0.001), Day 4 (median = 1.0 bees, *p* < 0.001), Day 5 (median = 2.0 bees, *p* < 0.001), and Day 6 (median = 2.0 bees, *p* < 0.001). Control bees also showed temporal changes (χ^2^ = 38.37, *p* < 0.001) but maintained consistently higher indoor counts throughout the observation period. Between-group comparisons revealed significant differences at all time points (Mann–Whitney *U*-tests, all *p* < 0.001), with effect sizes ranging from 0.70 to 1.00, indicating large treatment effects.

Seven-day-old bees monitored for 10 days post-injection showed the most pronounced and sustained response to melatonin treatment (Figure 1B). Melatonin-treated bees demonstrated significant temporal changes in indoor counts (χ^2^ = 170.29, *p* < 0.001), with counts dropping sharply from Day 1 (median = 8.0 bees) to Day 2 (median = 6.0 bees, *p* = 0.005) and reaching minimal levels by Day 3 (median = 2.0 bees, *p* < 0.001). Indoor activity remained consistently low through Day 10 (median = 1.0 bees). Control bees also showed temporal variation (χ^2^ = 87.73, *p* < 0.001) but maintained substantially higher indoor counts throughout the study period. Treatment effects were significant at all time points (all *p* < 0.001), with effect sizes of 0.61–1.00, demonstrating consistent large effects of melatonin across the extended observation period.

Fifteen-day-old bees monitored for 5 days post-injection exhibited significant temporal changes in both treatment groups (melatonin: χ^2^ = 62.15, *p* < 0.001; control: χ^2^ = 57.86, *p* < 0.001; Figure 1C). In melatonin-treated bees, indoor counts declined progressively from Day 1 (median = 4.5 bees) to near-zero levels by Days 4–5 (median = 0.0 bees for both days, *p* < 0.001). Control bees showed a more gradual decline but maintained higher indoor activity throughout. Significant between-group differences were observed at all time points (all *p* < 0.01), with effect sizes ranging from 0.60 to 0.96.

To assess whether reduced indoor activity corresponded to accelerated behavioral development, we measured the time to first waggle dance performance, a key milestone in foraging ontogeny. Melatonin treatment significantly reduced the time to first waggle dance compared to controls (Figure 1D). Melatonin-treated bees performed their first waggle dance at a median of 11.8 days (IQR: 9.8–14.0 days), compared to 20.9 days (IQR: 18.8–26.0 days) in control bees (Mann–Whitney *U*-test: U = 18.5, *p* < 0.001). The effect size was large (Common Language Effect Size = 0.94), indicating that 94% of melatonin-treated bees initiated waggle dancing earlier than control bees. This represents an approximate 9-day acceleration in the onset of this key foraging behavior.

These results demonstrate that a single melatonin injection consistently reduces indoor hive activity across different bee age groups and accelerates the transition to foraging behavior, suggesting a fundamental role for melatonin in regulating honey bee behavioral development and division of labor.

### 2.2. Behavioral Changes in Foragers Injected with Luzindole, a Melatonin Receptor 2 Antagonist

**Figure 2:**
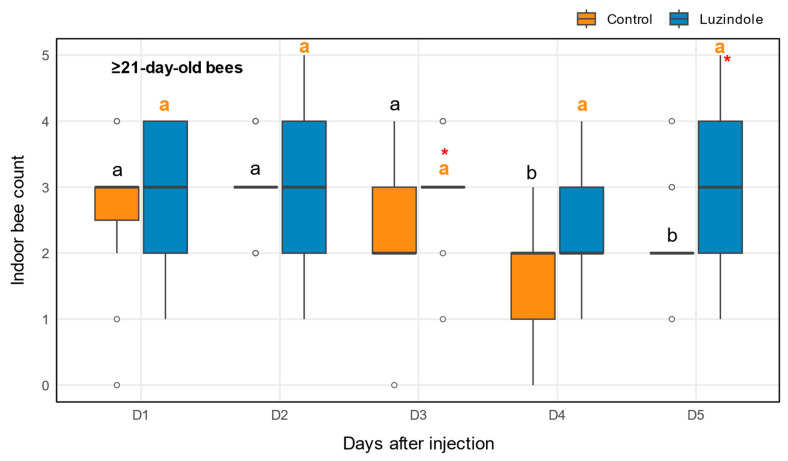
Luzindole injection inhibits foraging activity, as measured by increased hive retention. The bar graph shows the mean number of bees that did not forage (remained inside the hive) on days 1–5 after injection with 100 ng luzindole or a vehicle control (*n* = 15 per group). Data are mean ± SD. Open circles represent statistical outliers. Significant differences over time within each treatment group are indicated by different letters (orange for luzindole, black for control; Friedman test with Nemenyi post hoc, *p* < 0.05). Significant differences between treatment groups on individual days are marked by an asterisk (Mann–Whitney *U*-test, *p* < 0.05).

To further investigate the role of melatonin signaling in honey bee foraging behavior, we examined the effects of luzindole, a melatonin receptor antagonist, on hive retention in mature (≥21-day-old) forager bees. We monitored the number of bees that remained inside the hive (non-foraging bees) over a 5-day period following injection with 100 ng luzindole or vehicle control (Figure 2).

Luzindole-treated bees exhibited remarkably stable indoor bee counts throughout the observation period, with no significant temporal changes detected (Friedman test: χ^2^ = 5.58, *p* = 0.23; Figure 2). Indoor counts remained consistent across all days, with median values ranging from 2.0 to 3.0 bees and minimal variation (Day 1: 3.0 [IQR 2.0–4.0]; Day 5: 3.0 [IQR 2.0–4.0]). In contrast, control bees demonstrated significant temporal changes in indoor activity (χ^2^ = 22.36, *p* < 0.001), following a pattern consistent with natural foraging progression in mature bees. Control bees showed a significant decline from Day 2 (median = 3.0 bees [IQR: 3.0–3.0]) to Day 4 (median = 2.0 bees [IQR: 1.0–2.0], *p* = 0.002) and Day 5 (median = 2.0 bees [IQR: 2.0–2.0], *p* = 0.04). Additionally, indoor counts on Day 1 were significantly higher than Day 4 (*p* = 0.04).

Between-group comparisons revealed significant differences at two critical time points. On Day 3, luzindole-treated bees showed significantly higher indoor counts compared to controls (Mann–Whitney *U*-test: U = 156, *p* = 0.049, effect size = −0.38), with luzindole bees maintaining higher hive retention (median = 3.0 bees) versus controls (median = 2.0 bees). This difference became more pronounced by Day 5, where luzindole treatment resulted in significantly higher indoor counts (U = 160, *p* = 0.033, effect size = −0.42) compared to controls (median = 3.0 vs. 2.0 bees, respectively).

No significant differences were observed between groups on Days 1, 2, and 4 (all *p* > 0.05), indicating that the effects of luzindole became apparent as the observation period progressed.

### 2.3. MTR Knockdown and Foraging Behavior

**Figure 3:**
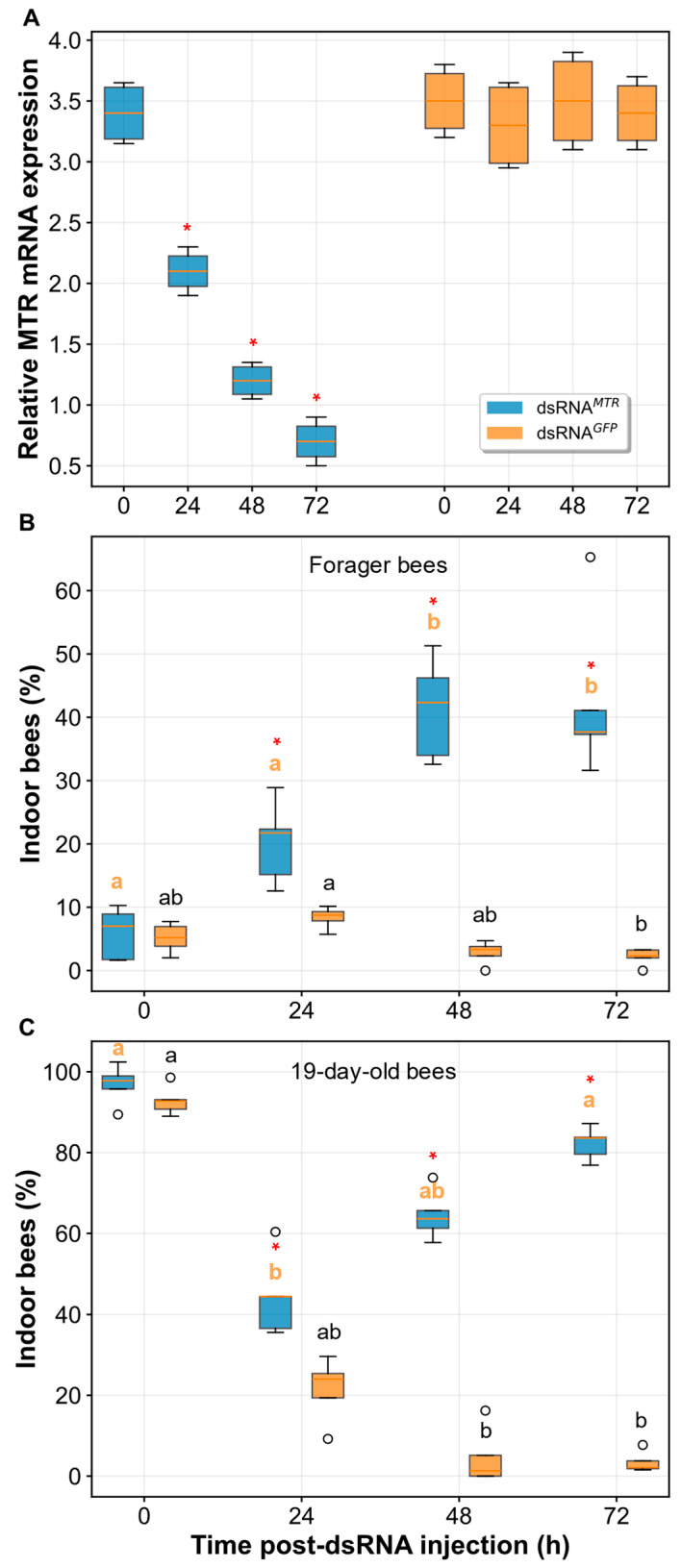
RNAi-mediated knockdown of the melatonin receptor gene (*AmMTR*/*AmMT2*) and its effect on foraging behavior. (**A**) Efficiency of *AmMTR* transcript suppression following dsRNA injection. (**B**,**C**) Impact of *AmMTR* knockdown on hive retention behavior in (**B**) forager-age bees and (**C**) 19-day-old bees after injection with dsRNA-*MTR* or control dsRNA-*GFP*. Data represent mean ± SD. Open circles represent statistical outliers. Within each treatment group, significant differences over time are indicated by different lowercase letters (orange letters: dsRNA-*MTR*; black letters: dsRNA-*GFP* control; Friedman test with Nemenyi post hoc, *p* < 0.05). Asterisks (*) denote a significant difference from the control group at a specific time point (Mann–Whitney *U*-test, *p* < 0.05).

To directly test the role of melatonin receptor signaling in honey bee foraging behavior, we employed RNA interference (RNAi) to knock down expression of the melatonin receptor gene (*AmMTR*/*AmMT2*). dsRNA-*MTR* injection achieved significant and progressive suppression of *MTR* transcript levels compared to dsRNA-*GFP* controls (Figure 3A). At baseline (0 h), no significant difference in *MTR* expression was detected between treatment groups (Mann–Whitney *U*-test: U = 11.5, *p* = 0.38). However, *MTR* expression was significantly reduced in dsRNA-*MTR*-treated bees by 24 h post-injection (median = 2.1 vs. 3.3 in controls; U = 16.0, *p* = 0.029) and remained suppressed through 48 h (median = 1.2 vs. 3.5 in controls; U = 16.0, *p* = 0.029) and 72 h (median = 0.7 vs. 3.4 in controls; U = 16.0, *p* = 0.029), representing approximately 65%, 66%, and 79% reductions in *MTR* expression at these respective time points.

Following successful *MTR* knockdown, we examined the effects on foraging behavior by measuring hive retention (percentage of indoor bees) in mature forager bees over a 72 h period (Figure 3B). dsRNA-*MTR*-treated foragers showed significant temporal changes in indoor activity (Friedman test: χ^2^ = 13.56, *p* = 0.004), with indoor bee percentages increasing progressively from 1 h (median = 7.0% [IQR: 1.7–8.9%]) to 48 h (median = 42.3% [IQR: 34.0–46.2%], *p* = 0.017) and 72 h (median = 37.7% [IQR: 37.3–41.1%], *p* = 0.008). In contrast, dsRNA-*GFP* control foragers exhibited different temporal dynamics (χ^2^ = 10.68, *p* = 0.014), with indoor percentages remaining consistently low throughout most of the observation period, showing only a significant decrease from 24 h (median = 8.8% [IQR: 7.8–9.3%]) to 72 h (median = 2.3% [IQR: 2.0–3.2%], *p* = 0.008). Between-group comparisons revealed no significant difference at 1 h post-injection (*p* = 0.84), but *MTR* knockdown resulted in dramatically higher hive retention at all subsequent time points: 24 h (21.7% vs. 8.8%, *p* = 0.008, effect size = −1.00), 48 h (42.3% vs. 3.3%, *p* = 0.008, effect size = −1.00), and 72 h (37.7% vs. 2.3%, *p* = 0.008, effect size = −1.00). The large effect sizes (1.00) indicate complete separation between treatment groups at these time points.

To determine whether *MTR* knockdown affects bees at different developmental stages, we examined 19-day-old bees, which represent the pre-forager to early forager transition period (Figure 3C). Both treatment groups showed significant temporal changes (dsRNA-*MTR*: χ^2^ = 15.00, *p* = 0.002; dsRNA-*GFP*: χ^2^ = 14.04, *p* = 0.003), but with markedly different patterns.

dsRNA-*MTR*-treated bees maintained high indoor percentages initially (0 h: median = 97.8% [IQR: 95.8–99.0%]) and showed a significant decrease by 24 h (median = 44.4% [IQR: 36.6–44.4%], *p* = 0.001), but then indoor percentages increased again at 48 and 72 h (63.6% and 83.6%, respectively). dsRNA-*GFP* controls showed a dramatic decline in indoor activity from baseline (median = 92.8% [IQR: 90.8–93.1%]) to 48 h (median = 1.3% [IQR: 0.0–5.1%], *p* = 0.003) and 72 h (median = 2.1% [IQR: 1.9–3.8%], *p* = 0.036), representing the expected transition from indoor to foraging activities. Between-group differences were not significant at baseline (*p* = 0.22) but became highly significant from 24 h onward: 24 h (44.4% vs. 24.0%, *p* = 0.012, effect size = −1.00), 48 h (63.6% vs. 1.3%, *p* = 0.012, effect size = −1.00), and 72 h (83.6% vs. 2.1%, *p* = 0.008, effect size = −1.00).

### 2.4. Quantification of α-Glucosidase mRNA as an Internal Work Class Marker in the Brain of Apis mellifera After Adult Emergence

**Figure 4:**
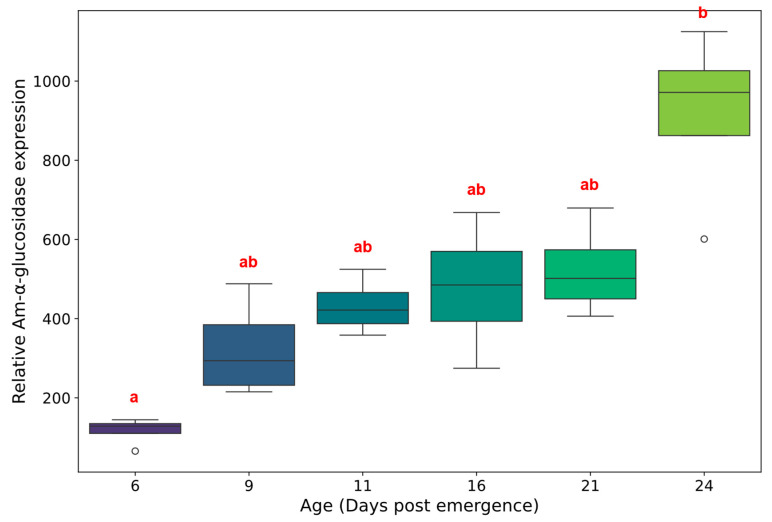
Age-dependent expression of *Amα-glucosidase* in the honey bee brain. Relative mRNA levels of *Amα-glucosidase* were quantified by qRT-PCR in worker bees at 6, 9, 11, 16, 21, and 24 days after adult emergence. Data represent mean ± SD. Open circles represent statistical outliers. Significant differences in transcript levels across age groups are indicated by different lowercase letters (Kruskal–Wallis test with Dunn’s post hoc test for pairwise comparisons, Bonferroni corrected, *p* < 0.05).

To investigate potential molecular markers associated with foraging behavior development, we examined the age-dependent expression pattern of *Amα-glucosidase*, a gene previously associated with foraging activity in honey bees. Brain tissue was collected from worker bees at six developmental time points representing the typical transition from in-hive to foraging activities (6, 9, 11, 16, 21, and 24 days post-emergence), and relative mRNA expression levels were quantified using qRT-PCR (Figure 4).

*Amα-glucosidase* expression showed significant age-dependent changes across the developmental time course (Kruskal–Wallis test: H = 17.68, *p* = 0.003). The effect size was large (η^2^ ≈ 0.70), indicating that age accounts for approximately 70% of the variance in *Amα-glucosidase* expression levels. Expression levels showed a general upward trend with age, beginning at the lowest levels in 6-day-old bees (median = 128.4, range: 65.4–144.5) and reaching peak expression in 24-day-old bees (median = 971.4, range: 601.0–1124.9). Intermediate age groups (9, 11, 16, and 21 days) showed progressively increasing median expression levels (293.7, 421.6, 485.0, and 501.6, respectively), though with considerable individual variation within each age group.

Post hoc pairwise comparisons using Dunn’s test with Bonferroni correction revealed one significant difference: 24-day-old bees showed significantly higher *Amα-glucosidase* expression compared to 6-day-old bees (*p* = 0.001). This represents approximately a 7.6-fold increase in median expression levels between the youngest and oldest age groups examined. Despite the clear overall age-related trend, other pairwise comparisons did not reach statistical significance after multiple comparison correction, likely due to the conservative Bonferroni adjustment and the substantial within-group variation observed, particularly in older age groups.

### 2.5. Neuromodulatory Regulation of Am-α-Glucosidase Expression

**Figure 5:**
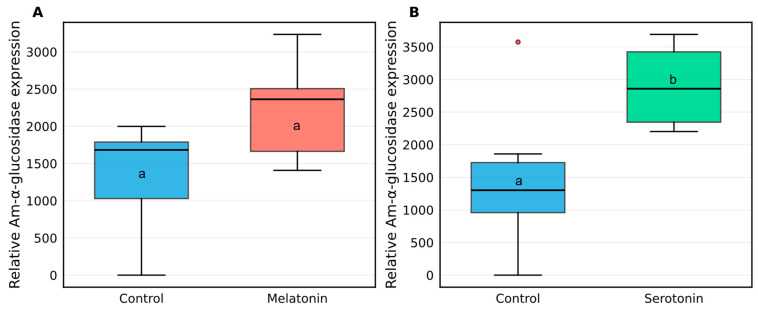
Neuromodulatory control of *Amα-glucosidase* expression in the honey bee brain. (**A**) Melatonin induces *Amα-glucosidase* expression in 7-day-old worker bees 24 h after injection with 100 ng melatonin. (**B**) Serotonin (5-HT) enhances *Amα-glucosidase* transcript levels in 23-day-old forager brains following a single hemocoelic injection of 1 pmol (in 5 µL dH_2_O). Data are presented as mean ± SD. Different lowercase letters indicate a significant difference (Mann–Whitney *U*-test, *p* < 0.05). Open circles (including the red circle in panel (**B**)) represent statistical outliers.

To investigate the potential neuromodulatory control of *Am-α-glucosidase* expression in honey bee brains, we examined the effects of melatonin and serotonin treatments on enzyme transcript levels (Figure 5).

Melatonin injection (100 ng) in 7-day-old worker bees resulted in elevated *Am-α-glucosidase* expression 24 h post-treatment compared to control bees (Figure 5A). The melatonin-treated group showed a mean relative expression of 2225.37 ± 694.98, while the control group exhibited 1333.00 ± 766.78. Despite this apparent increase in *Am-α-glucosidase* expression, Mann–Whitney *U*-test analysis revealed no statistically significant difference between the groups (U = 28.0, *p* = 0.132). The effect size was large (Common Language Effect Size = 0.778), indicating a 77.8% probability that a randomly selected observation from the melatonin group would exceed one from the control group.

In contrast, serotonin (5-HT) treatment demonstrated a significant regulatory effect on *Am-α-glucosidase* expression in 23-day-old forager brains (Figure 5B). Following hemocoelic injection of 1 pmol serotonin, treated bees showed significantly higher *Am-α-glucosidase* transcript levels (2899.60 ± 640.99) compared to controls (1481.58 ± 1198.28; U = 31.0, *p* = 0.041). This represents a statistically significant upregulation of *Am-α-glucosidase* expression in response to serotonin treatment. The effect size was large (Common Language Effect Size = 0.861), with an 86.1% probability that serotonin-treated bees would show higher expression than control bees.

### 2.6. Change in NAT Transcript During Adult Life

**Figure 6:**
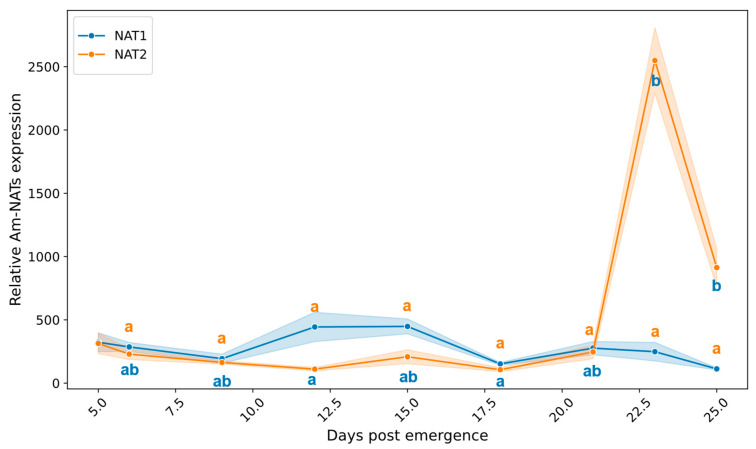
Age-related expression profiles of *AmNAT1* and *AmNAT2* in the worker honey bee. Relative mRNA levels of *AmNAT1* and *AmNAT2* were quantified by qRT-PCR across post-emergence developmental stages (days 3, 6, 9, 12, 15, 18, 21, 23, and 25). Data represent mean ± SD. Significant differences in transcript abundance across age groups for each gene are indicated by distinct lowercase letters (Kruskal–Wallis test with Dunn’s post hoc comparisons, Bonferroni corrected, *p* < 0.05; orange letters: *AmNAT1*; blue letters: *AmNAT2*).

To examine the developmental regulation of arylalkylamine *N*-acetyltransferase (NAT; typically referred to as AANAT) genes throughout the worker honey bee lifespan, we analyzed the expression profiles of *AmNAT1* and *AmNAT2* across nine time points from 5 to 25 days post-emergence (Figure 6).

*AmNAT1* showed relatively stable expression levels across all developmental stages examined (Kruskal–Wallis H = 13.37, *p* > 0.05). Expression levels ranged from 112.48 ± 17.01 in 25-day-old bees to 448.13 ± 118.18 in 15-day-old bees, but these differences were not statistically significant. *AmNAT2* displayed a dramatically different age-related expression pattern, characterized by a pronounced peak in forager-aged bees (Kruskal–Wallis H = 27.26, *p* < 0.001). The most striking feature was the massive upregulation at day 23, where expression levels reached 2549.38 ± 519.00, representing approximately a 10-fold increase compared to most other age groups. Expression then declined substantially by day 25 (912.95 ± 298.90). Post hoc comparisons revealed that 23-day-old bees showed significantly higher *AmNAT2* expression compared to 12-day-old (*p* = 0.005) and 18-day-old bees (*p* = 0.005), with strong statistical trends compared to other younger age groups.

### 2.7. Circadian Cis-Regulatory Landscape of the Honey Bee MTR Promoter

**Figure 7:**
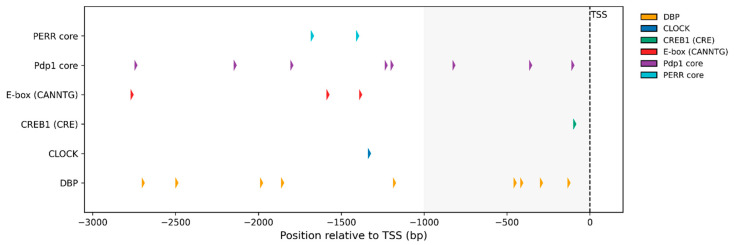
Distribution of circadian cis-regulatory motifs in the *Apis mellifera MTR* promoter. Predicted motifs within the 3 kb region upstream of the transcription start site (TSS; NC_037647.1:5,949,179) are shown as colored arrows: DBP/D-box (orange), CLOCK E-box (blue), CREB1/CRE (green), degenerate E-boxes (red), Pdp1 cores (purple), and PER-repeat cores (turquoise). A total of 23 sequence-verified sites were identified (11 PWM-based and 12 consensus/regex-based), with several promoter-proximal Pdp1 cores (−820, −357, and −102 bp) indicating a strong potential for circadian transcriptional regulation.

Predicted circadian cis-elements within the *A. mellifera MTR* promoter are summarized in Table S1 and their spatial distribution is illustrated in Figure 7. The PWM-based analysis identified 11 sequence-verified high-confidence motifs across the −3 kb promoter, including multiple DBP/D-box–like sites distributed from the distal (−2701, −2498 bp) to the proximal promoter (−457 to −132 bp), a CLOCK-like E-box at −1334 bp, and a CRE-like element only 93 bp upstream of the TSS. In parallel, the consensus/regex scan detected 12 exact motif cores, notably three clustered Pdp1 sites at −820, −357, and −102 bp, as well as degenerate E-boxes at −2762, −1580, and −1383 bp. Collectively, the *MTR* promoter contains multiple high-confidence PWM matches and consensus motifs—including DBP/D-box, Pdp1, CRE, and E-box elements—associated with circadian regulators, highlighting a strong potential for circadian transcriptional regulation of *MTR*; however, formal enrichment testing and experimental validation are needed to confirm functional regulatory activity.

### 2.8. Refined Structural Model of Apis mellifera MT2 Reveals Conserved Class A GPCR Architecture and Melatonin/Analog Binding Determinants

The best models of the *A. mellifera* MT2 receptor were valid based on the Ramachandran map (Table 1).

**Figure 8:**
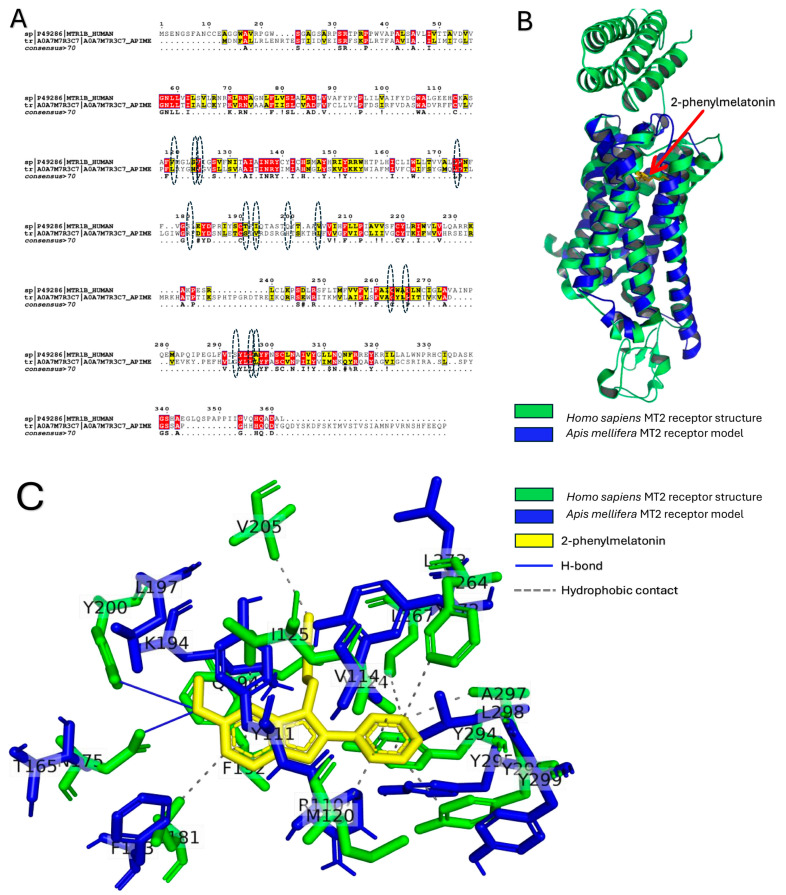
Comparative sequence and structural analysis of human and *Apis mellifera* MT2 receptors. The sequence (**A**) and structural (**B**) alignment between *Homo sapiens* (green) and *A. mellifera* (blue) MT2 receptors. The predicted binding site based on sequence and structural alignment are encircled. In the sequence alignment (**A**), residues that are identical across species are highlighted in red, while residues with high similarity are highlighted in yellow. The consensus/annotation line beneath the alignment uses the following symbols: . = weakly similar substitution. Program-specific annotation flags appearing in this alignment are defined here for clarity: # = alignment column flagged as low-occupancy or low-confidence (many gaps/poor support); $ = residue flagged for additional annotation (e.g., non-standard residue, potential post-translational modification, or program note); ! = position flagged for manual inspection or sequence conflict. These flags are generated by the alignment/visualization tool. (**C**) Structural superposition of the Galaxy-refined *A. mellifera* MT2 model (colored) with the human MT2 crystal structure (gray; PDB 6ME6). Models were aligned in PyMOL (version 3.1.6.1) (global Cα RMSD ≈ 2.31 Å). Conserved class A GPCR activation motifs (DRY, CWxP, NPxxY) are highlighted and labeled, and the extracellular loop topology—including the distinctive extracellular expansion of the bee ligand-binding pocket—is shown as a semi-transparent surface (inset). Key ligand-contact residues identified by docking (K194, K279, L298) are displayed as sticks. Scale bar = 10 Å.

Pairwise sequence alignment of the *Homo sapiens* and *A. mellifera* MT2 receptors is shown in Figure 8A by ESPript 3.0. The percent identity is only 6.35% between the two sequences despite regions of high similarity (highlighted in yellow) and identity (highlighted in red).

Structural alignment was performed between the *A. mellifera* MT2 receptor model and *H. sapiens* MT2 receptor structure (PDB ID: 6ME6) (see Figure 8B). The superposition root-mean square difference was only 2.306 Å (1228 atoms). The binding pose of the *H. sapiens* MT2 receptor to 2-phenylmelatonin is depicted in Figure 8C with the aid of PLIP webserver. The ligand is shown in orange sticks while the interacting residues are in cyan sticks. Three H-bonds are formed here; N175, Q194, and Y200. Twelve hydrophobic contacts are the main interaction theme of 2-phenylmelatonin to *H. sapiens* MT2 receptor; M120, V124, I125, L181, F192(2), V205, W264, L267, Y294, A297, and Y298. The dashed eclipses in Figure 8A represent the structural alignment residues that could be the binding region to melatonin and 2-phenylmelatonin. These residues are either identical or similar, indicating their potential binding site residues. This will be tested in the docking experiment as the next step.

**Figure 9:**
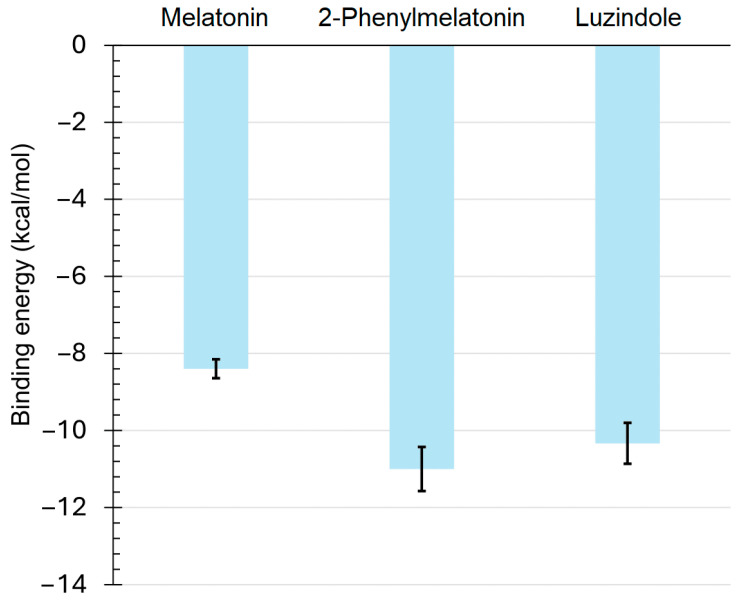
The average binding energies (kcal/mol) of the binding of melatonin, 2-phenylmelatonin, and luzindole against *Apis mellifera* MT2 receptor. Values are shown in kcal/mol. Error bars represent the standard deviation.

The molecular docking testing were conducted for three models of the *A. mellifera* MT2 receptor. The average binding energies (kcal/mol) were depicted in Figure 9. The binding was tighter for 2-phenylmelatonin, and luzindole compared to melatonin against *A. mellifera* MT2 receptor.

**Figure 10:**
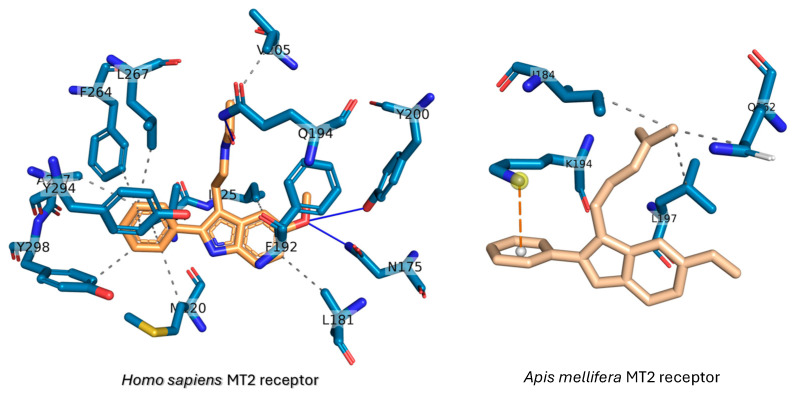
The formed interactions between 2-phenylmelatonin against *Homo sapiens* (**left**) and *Apis mellifera* (**right**) MT2 receptors. The protein residues are depicted in cyan sticks while the 2-phenylmelatonin is shown in wheat sticks. H-bonds are shown as blue lines, while dashed gray lines and dashed orange lines are hydrophobic contacts and π-cation interactions, respectively.

The details of the formed interactions are listed in Table 2. The main types of interactions are hydrophobic contacts, while few interactions are due to π-cation and H-bonding. The main residues that take part in the interactions are L298 (Melatonin and luzindole), K194 (2-phenylmelatonin), and K279 (luzindole). Figure 10 shows the detailed interactions that formed upon docking the melatonin, 2-phenylmelatonin, and luzindole against *A. mellifera* MT2 receptor.

Docking (Figure 10) shows 2-phenylmelatonin and luzindole bind more tightly than melatonin and that interactions are dominated by hydrophobic contacts (notably L298 and neighboring residues), with ligand-specific contributions from K194 and K279 (π-cation/H-bonding), consistent with SAR observed for human MT2.

Overall, PHYRE2/Galaxy-refined *A. mellifera* MT2 models are stereochemically robust (Ramachandran favored ≈98.6%) and superpose on human MT2 (PDB 6ME6) with an RMSD of ~2.31 Å despite low sequence identity. Docking (AutoDock Vina, exhaustiveness 64) predicts stronger binding for 2-phenylmelatonin and luzindole than for melatonin; interactions are primarily hydrophobic with key ligand-contact residues L298, K194 and K279 identified across models. These findings support a conserved melatonergic binding mode with insect-specific extracellular pocket topology.

## 3. Discussion

Our results identify melatonergic signaling as a circadian-linked modulator of behavioral maturation in *A. mellifera*. A single hemocoelic melatonin dose reduced hive retention and advanced first waggle dance by ≈9 days (median 11.8 vs. 20.9 days; CLES = 0.94), while luzindole antagonism and RNAi knockdown of a putative melatonin receptor (*AmMTR*/*AmMT2*) produced reciprocal suppression of foraging.

The classic JH–Vg double-repressor model explains many maturation features but is insufficient alone (e.g., brood-deprived nurses can become rhythmic/forage without a JH surge [18,22]). Biogenic amines (OA, dopamine, 5-HT) also bias responsiveness and drive precocious foraging [7,26,50]. Our data extend these frameworks by showing that melatonin provides an orthogonal, clock-linked layer that tunes timing of maturation: acute melatonergic activation advances circadian-structured foraging, whereas receptor antagonism stabilizes intranidal behavior, complementing rather than replacing JH–Vg and amine pathways [2,3,28].

Three lines of evidence support melatonin as a “gatekeeper”. First, behavioral phenotypes were robust across ages but strongest when melatonin was applied near the mid-nurse window (e.g., 7-day cohort), consistent with advancing the onset of circadianized extranidal activity [34,51,52]. Second, luzindole stabilized forager indoor counts, indicating intact melatonergic signaling helps maintain the outward bias typical of foragers (paralleling photoperiodic effects in other insects [39]). Third, endogenous biosynthesis (*AmNAT2*) peaks in foragers (~day 23), supporting the idea that endogenous melatonin production scales with maturation [34,38].

Based on this convergence of evidence, we propose the following testable mechanistic hypothesis: circadian-driven expression of *AmMT2* in key brain regions (e.g., mushroom body Kenyon cells) enhances sensitivity to melatonin, which subsequently potentiates octopaminergic signaling in circuits governing sucrose responsiveness (e.g., the antennal lobe), thereby lowering the neural threshold for initiating foraging behavior.

Behavioral manipulations converge with molecular and structural data: RNAi reduced *AmMTR* mRNA (~65–79% at 24–72 h) and suppressed foraging, establishing receptor-dependence; atomistic modeling classifies AmMTR as a class-A GPCR that superposes on human MT2 (PDB 6ME6) and predicts melatonin-compatible pockets with recurring contact residues (e.g., L298, K194, K279) despite low sequence identity and template-dependent uncertainty [47,53]. These in silico results are hypothesis-generating and define clear targets for biochemical validation. Complementary promoter analyses identify canonical circadian motifs (E-boxes, D-boxes, CRE, and Pdp1-like cores) upstream of *AmMTR*, indicating potential clock-related regulation [54,55] and providing a mechanistic route by which circadian transcription could regulate receptor availability and thus behavioral state.

Our findings are compatible with, and likely intersect, amine-mediated motivational systems. OA enhances sucrose responsiveness and can induce precocious foraging [7,50]; dopamine rises during dance behavior and modulates reward-related signaling [56]. 5-HT is the direct biosynthetic precursor of melatonin [35,36]. We observed a significant 5-HT-driven upregulation of brain *Amα-glucosidase* (a forager marker; [9,14,15]), whereas melatonin produced a large, non-significant trend in the same direction—consistent with a 5-HT → melatonin axis that shapes metabolic readiness. Definitive resolution will require co-manipulation experiments across defined age/classes and simultaneous readouts of clock gene expression, receptor abundance, and amine titers.

Metabolically, foraging entails a shift toward carbohydrate utilization and increased expression of honey-processing enzymes such as α-glucosidase in the brain and HPGs [7,9]. The melatonin-evoked behavioral acceleration coincided with transcriptional signatures consistent with this metabolic reorientation. In light of transcriptional reprogramming observed during maturation (e.g., *Broad Complex* dynamics; [2]) and emerging lipidomic remodeling across tasks [3], a parsimonious interpretation is that melatonin contributes to aligning neuromodulatory tone with cellular metabolic state as circadian control is established.

While our findings demonstrate that melatonin can modulate the timing of behavioral maturation, we do not interpret it as a primary or standalone regulator of task allocation. Instead, we view melatonin as acting alongside the established JH–Vg axis and biogenic amine pathways that also shape polyethism. Additional colony-level replication and direct receptor-level functional validation will be important for defining the broader generality of this pathway.

A key temporal feature of our data is that melatonin’s effects unfold over days and luzindole’s suppression of foraging is similarly gradual, consistent with melatonin acting as a circadian-informed instructive signal rather than an acute locomotor stimulant. The detection of circadian cis-elements near *AmMTR* and the developmental peak in *AmNAT2* support a model in which the 5-HT → AANAT → melatonin pathway relays time-of-day and age information to neural and endocrine effectors; AANAT-driven melatonin rhythms regulate photoperiodic and neuroendocrine outputs in insects [39,46], and analogous mechanisms likely underlie the transition from arrhythmic nursing to strongly rhythmic foraging in honey bees [29,32,34]. Because injections were given in the early evening (18:00 to 19:00), when clock output changes rapidly, sensitivity may be phase-dependent and explicit time-of-day response curves for both agonists and antagonists are needed.

Alternative explanations remain: melatonin’s pleiotropy (antioxidant, immunomodulatory) could improve physiological condition rather than convey circadian information [42,57,58], but three observations argue against a purely tonic effect—(i) reciprocal suppression by a receptor antagonist, (ii) parallel suppression after receptor knockdown, and (iii) structural/promoter evidence for a bona fide, clock-linked melatonin receptor. Off-target actions of luzindole or dsRNA cannot be fully excluded; we reduced this risk by targeting *AmMTR*/*AmMT2* and confirming knockdown outside the dsRNA region, yet CRISPR/Cas rescues (e.g., luzindole-insensitive receptor variants) would strengthen causal inference. We also did not quantify endogenous melatonin titers, so longitudinal—ideally single-bee—measures of melatonin, *AmNAT* isoforms and *AmMTR* across maturation (building on prior reports of higher melatonin in forager brains) are essential to relate effect sizes to physiological concentrations and colony context [34,38].

Methodological choices frame interpretation. Hemocoelic injection gives systemic exposure but does not localize action, so neuroanatomical receptor mapping (antennal lobes, mushroom bodies, central complex, periphery) and higher-resolution behavioral telemetry are necessary to define circuit-level mechanisms and decompose effects on bout structure, navigation and reward learning. Ecologically, melatonin’s ties to biogenic-amine systems imply vulnerability to sublethal agrochemical perturbation of amine balance or energy reserves, which could shift melatonin-tuned thresholds for foraging and disrupt task allocation without causing overt mortality [59,60]. From an evolutionary perspective, AANAT-mediated melatonin synthesis is an ancient timing mechanism repeatedly co-opted for life-history transitions (e.g., diapause) [39,61]; the identification of a structurally credible, clock-linked AmMTR whose expression affects foraging suggests social insects may have recruited this conserved indolamine pathway to align colony labor with diel cycles, with melatonin’s protective roles potentially supporting resilience under environmental stressors [62].

We propose the following working model. As workers age, *AmNAT2* expression increases, elevating melatonin production in brain regions implicated in sensory integration and motivation. Circadian transcription factors modulate *AmMTR* availability, creating time-of-day windows during which melatonin more effectively biases neural circuits toward extranidal activity. In parallel, OA and dopamine adjust responsiveness to reward and sensory cues [50,56]; Vg–JH governs systemic physiological state (e.g., HPGs regression; [6]). Melatonin therefore functions as a clock-synchronized permissive signal that reduces the threshold for the initiation and maintenance of foraging behavior, whereas pharmacological antagonism or receptor knockdown abolishes this permissive effect, thereby stabilizing intranidal behavior. This integrative model parsimoniously explains the asymmetric effects of our perturbations and accommodates prior observations of circadian maturation [16,63], amine-driven precocity [7,26], and JH–Vg plasticity [18,20].

Two priorities emerge. Mechanistically, neuroanatomical mapping of *AmMTR* and circuit-level manipulations (e.g., targeted RNAi/CRISPR, opto/chemogenetics where feasible) should identify sites of action and interactions with OA and dopamine pathways. Ecologically, field experiments that modulate melatonergic tone (feeding, antagonism) across seasons, nutritional states, and agrochemical exposures are needed to quantify the contribution of this pathway to colony performance and resilience. Integrating these approaches with time-of-day dependent designs and high-resolution behavioral telemetry will clarify how melatonin—and its receptor—shape the temporal control of social behavior. While the convergent evidence is compelling, receptor-level functional assays and expanded colony replication are required to confirm generality and mechanistic causality.

## 4. Materials and Methods

### 4.1. Honey Bee Colonies and Marking Procedure

Colonies of Western honey bees (*A. mellifera*) were purchased from Mamuro Bee Farm (Saitama Prefecture, Japan) and housed in observation hives at Kobe University Experimental Garden under natural outdoor conditions. Each colony contained a mated queen and a full complement of worker bees, with colony sizes ranging from 15,000 to 20,000 individuals. Newly emerged adult bees were collected daily from the brood frames, immobilized briefly on ice for approximately 1–2 min to facilitate accurate marking, and individually tagged with numbered plastic tags or enamel paint applied to the dorsal side of the thorax. The marked bees were then reintroduced into their respective hives and allowed a 24 h acclimation period prior to experiments. Throughout the experimental period, marked bees had ad libitum access to a sucrose solution (1:1 *w*/*v*) and water.

### 4.2. Pharmacological and RNAi Injections

Bees of known ages were collected from the observation hive, briefly immobilized on ice, and injected via the intersegmental membrane of the thorax using a Hamilton microsyringe (Hamilton Company, Reno, NV, USA). To assess the effect of melatonin on nursing bees, two-, seven-, and 15-day-old bees (*n* = 24, 30, and 18, respectively) received a single 1 µL injection of 100 ng melatonin (Wako Chemicals, Osaka, Japan) dissolved in 1% ethanol (*v*/*v*) or vehicle control (1% ethanol). To investigate the role of melatonin signaling in foragers, mature bees (≥21 days old, *n* = 15) were injected with 100 ng of the melatonin receptor antagonist luzindole (Sigma Aldrich, St. Louis, MO, USA) in 1% DMSO; control foragers (*n* = 15) received 1% DMSO alone. Injections were administered for four consecutive days between 18:00 and 19:00 h. To examine the effect of serotonin on gene expression, 23-day-old forager bees (*n* = 10) received a single 5 µL injection of 1 pmol serotonin (5-HT; Sigma Aldrich, USA) in distilled water, with controls receiving distilled water alone. Vehicle solvents were used at minimal concentrations and identical injection volumes across all treatments. Vehicle-injected bees did not display overt behavioral abnormalities during routine observation, and all pharmacological effects were analyzed relative to matched vehicle controls.

Dose selection and pharmacological considerations—The 100 ng per-bee dose for melatonin and the 100 ng per-bee dose for luzindole were chosen based on: (i) pilot dose–range experiments conducted in our laboratory that identified 100 ng as a dose producing consistent behavioral modulation without observable acute toxicity and (ii) doses commonly used in hemocoelic injection studies in insects where systemic access (rather than fine pharmacokinetics) is the experimental goal. Injections were delivered as 1 µL boluses between 18:00 and 19:00 to coincide with a period of rapid clock output change. We note that the present study did not quantify endogenous melatonin titers in individual bees; therefore, direct comparisons between the injected dose and physiological concentrations are not possible here and will be addressed in follow-up experiments.

### 4.3. Melatonin Receptor (MTR) Knockdown via RNA Interference (RNAi)

A putative melatonin receptor (*AmMTR*; LOC409159, XM_392683.6) was identified in the *A. mellifera* genome by BLAST (NCBI BLASTp/tBLASTn, version 2.13.0+, https://blast.ncbi.nlm.nih.gov/, accessed on 16 December 2025) searches using insect and vertebrate MTR protein sequences as queries. Because sequence similarity alone does not establish function, the receptor is referred to as a putative *AmMTR* pending functional validation. Evidence for receptor-mediated melatonin actions in insects has been reported [39,44,45,46], and this candidate receptor was therefore selected for downstream molecular interrogation. Templates for dsRNA synthesis were generated by PCR amplification of an internal *AmMTR* fragment using gene-specific primers with a T7 promoter sequence appended to both forward and reverse primers (Supplementary Table S2). PCR products were visualized on agarose gels, and correctly sized bands were purified using the GFX PCR DNA & Gel Band Purification Kit (GE Healthcare, Chicago, IL, USA). Double-stranded RNA was synthesized with the MEGAscript™ RNAi Kit (Ambion/Thermo Fisher, Waltham, MA, USA) following the manufacturer’s instructions; reactions were DNase-treated, cleaned, quantified, and integrity verified by agarose electrophoresis. Non-targeting *GFP* dsRNA was prepared using the same T7-tailed strategy [39]. Immediately prior to injection, dsRNA was mixed 1:1 (*v*:*v*) with Metafectene PRO (Biontex, Mainz, Germany) to enhance uptake as described by Mohamed et al. [39]. Forager bees (≥21 days old), identified by pollen loads at the hive entrance, were briefly anesthetized on dry ice, marked, and injected as described previously. Each bee received 2 µg dsRNA in 2 µL PBS (1 µg/day for two consecutive days); control bees received vehicle only (PBS ± Metafectene). Treated bees were monitored daily, and foraging suppression was assessed during peak colony activity (14:00–15:00). Brains (BR–SOG complexes) were dissected in ice-cold PBS (1×: 137 mM NaCl, 2.7 mM KCl, pH 7.4) and stored at −80 °C. Knockdown efficiency was assessed by qPCR at 0, 24, 48, and 72 h post-injection (see qPCR methods below).

### 4.4. Behavioral Assays

Behavioral data were collected manually or via video recording to ensure consistency and allow objective quantification of activity patterns. This approach aligns with established methodologies in honey bee research, including fine-grained behavioral tracking under controlled conditions [64].

#### 4.4.1. Indoor Hive Activity and Hive Retention

For melatonin, luzindole, and *MTR* knockdown experiments, indoor hive activity was quantified by recording the number or percentage of marked bees remaining inside the hive. Forager activity was assessed daily during the peak foraging window (14:00–15:00 h), a period characterized by stable and robust flight activity at the hive entrance. Nurse bee activity was monitored over several days post-injection as specified in the results.

#### 4.4.2. Onset of Foraging Behavior (Waggle Dance)

To assess if melatonin treatment accelerated behavioral development, the age at which bees performed their first waggle dance was recorded. Marked bees from the melatonin and vehicle control treatment groups were monitored daily, and the age of first waggle dance performance was noted for each individual.

### 4.5. Molecular Analyses

#### 4.5.1. RNA Extraction and cDNA Synthesis

Total RNA for cloning, dsRNA verification, and qPCR assays was extracted using RNAiso Plus (Takara, Shiga, Japan) according to the manufacturer’s protocol. Whole worker bee brains (BR–SOG complex where specified) were dissected in ice-cold PBS and homogenized immediately; developmental time-series samples were processed in the same way. RNA integrity was confirmed by agarose electrophoresis (distinct 28S and 18S rRNA bands), and purity was assessed by NanoDrop spectrophotometry (Thermo Fisher Scientific, Waltham, MA, USA) (A260/280 = 1.9–2.1). One microgram of total RNA was DNase-treated if required and reverse-transcribed using ReverTra Ace (Toyobo, Osaka, Japan) with a mixed priming strategy (oligo[dT]_18_ plus random hexamers). Reverse-transcription conditions were 65 °C for 5 min (denaturation), 37 °C for 15 min (primer annealing), 50 °C for 5 min (extension), and 98 °C for 5 min (enzyme inactivation), followed by a hold at 4 °C. cDNA was stored at −20 °C, and unless otherwise specified, 2.5 µL of diluted cDNA was used per 20 µL qPCR reaction.

#### 4.5.2. Quantitative Real-Time PCR (qPCR; General Procedures)

Quantitative PCR was performed on a Thermal Cycler Dice Real Time System (Takara) using THUNDERBIRD™ SYBR qPCR Mix (Toyobo). Each 20 µL reaction contained 1× SYBR mix, 200 nM of each primer, and 2 µL cDNA (≈40 ng RNA-equivalent). Cycling conditions were 95 °C for 1 min, followed by 40 cycles of 95 °C for 15 s and 60 °C for 1 min. Single-product amplification was confirmed by dissociation curve analysis (65–95 °C, +0.5 °C/s). Primer sets were designed for gene-specific amplification and run under qPCR conditions commonly used in honey bee studies and others [39,65,66]. Each sample was analyzed in technical triplicate; biological replicates consisted of independent pools of five brains unless otherwise indicated. Transcript levels were normalized to *rp49* (*RPL32*), and relative expression calculated using the 2^−ΔΔCt^ method [67]. For knockdown verification, primers amplified regions outside the dsRNA target sequence to avoid measurement artifacts (Supplementary Table S2).

#### 4.5.3. Quantification of Gene Expression by qPCR

Expression of α-*glucosidase* (*Hbg3*) and *AANAT* isoforms was quantified from total RNA extracted from worker bee brains (BR–SOG complex where indicated) and reverse-transcribed as described above. qPCR reactions followed the same procedures, with primer identifiers and amplicon sizes provided in Supplementary Table S2. Expression of *Hbg3* (NM_001040236) was examined across developmental stages (6–24 days post-emergence), while transcript levels of *AANAT1* (XM_026443226.1) and *AANAT2* (XM_026443227.1) were quantified using isoform-specific assays targeting unique 3′-UTR regions. Relative expression for all genes was calculated using the 2^−ΔΔCt^ method with efficiency correction.

### 4.6. Statistical Analysis

All statistical analyses were conducted using R statistical software (version 4.4.1; [68]), with statistical significance set at *p* < 0.05 for all tests. Preliminary assessment of data distribution using Shapiro–Wilk tests revealed that the majority of behavioral and gene expression data deviated from normality. Consequently, non-parametric statistical methods were employed throughout all analyses to ensure robust and appropriate statistical inference.

Temporal changes in daily foraging patterns, measured by indoor bee counts for each treatment group (melatonin, luzindole, and dsRNA-*MTR*), were assessed using the Friedman test followed by the Nemenyi post hoc test. Subsequent pairwise comparisons between each experimental treatment group and the control group at individual time points were performed using Mann–Whitney *U* tests. The time to first waggle dance performance was compared between melatonin-treated and control bees using a Mann–Whitney *U* test.

Clarification of repeated-measures design and statistical rationale—All behavioral metrics (indoor hive counts, hive-retention percentages, and age at first waggle dance) were recorded from individually marked workers that were followed after re-introduction to their observation hive; thus the behavioral time-series represent repeated measures on the same bees. Shapiro–Wilk tests indicated frequent departures from normality and group sizes were modest; consequently we used non-parametric repeated-measures tests that are robust given these data properties. Within-group temporal changes were analyzed with the Friedman test (the non-parametric analog of repeated-measures ANOVA) with Nemenyi post hoc comparisons. Pairwise between-group contrasts at individual timepoints were performed with Mann–Whitney *U* tests to provide clear, effect-size-oriented comparisons. Because multiple per-timepoint pairwise tests can increase Type-I error, we interpreted these contrasts conservatively, emphasizing results that were (i) supported by the overall Friedman test, (ii) consistent in direction with sizeable common-language effect sizes across adjacent timepoints, and (iii) concordant with independent manipulations (pharmacological antagonism and RNAi knockdown). Exact *p*-values and common-language effect sizes are reported for transparency. Behavioral experiments were conducted in observation hive(s); colony and hive handling details are provided in Section 4.1.

To validate gene knockdown efficiency, *MTR* mRNA expression levels following dsRNA-*MTR* injection were compared to those in dsRNA-*GFP* (control)-injected groups at individual time points using Mann–Whitney *U* tests. To evaluate the impact of neurochemicals on gene expression, the effects of melatonin and serotonin on *Amα-glucosidase* mRNA levels were assessed. Specifically, 7-day-old bees injected with 100 ng melatonin were compared to uninjected controls 24 h post-injection using a Mann–Whitney *U* test. Similarly, 23-day-old foragers injected with 1 pmol serotonin (in 5 µL dH_2_O) were compared to vehicle-injected controls (5 µL dH_2_O alone) using a Mann–Whitney *U* test. Finally, the effect of bee age on *Amα-glucosidase* and *AmNATs* expression levels was examined using Kruskal–Wallis tests. When a significant main effect was detected, Dunn’s post hoc tests with Bonferroni correction were conducted for pairwise comparisons between age groups.

### 4.7. In Silico Identification of Circadian Cis-Regulatory Motifs in the MTR Promoter

The 3 kb region upstream of the *A. mellifera* *MTR* transcription start site (TSS; NC_037647.1:5,949,179, Amel_HAv3.1) was scanned for candidate circadian cis-regulatory motifs using two complementary strategies. First, a PWM-based (FIMO-style) scan was performed with JASPAR motif profiles [69,70] for canonical circadian regulators (CLOCK/BMAL1 E-box, DBP D-box, CREB1 CRE, ROR elements). We retained high-confidence PWM matches using a relative PWM score threshold ≥ 0.85; all retained hits were re-verified against the genomic FASTA and reported as coordinates relative to the annotated TSS (negative values = upstream). Because this analysis was implemented as a focused PWM scoring procedure on the extracted promoter sequence rather than a genome-wide FIMO run, we did not report per-site FIMO *p*-values here. For reference, a conventional genome-wide FIMO approach would typically report per-site *p*-values (e.g., *p* ≤ 1 × 10^−4^) and q-values (FDR-corrected) computed against an explicit background nucleotide model (for example, a zero-order or first-order Markov model derived from a set of *A. mellifera* promoter sequences), which could be explored in future analyses.

Second, to capture canonical and degenerate short elements we carried out a consensus/regex search for established circadian motifs, including the canonical E-box (CACGTG), degenerate E-box (CANNTG), D-box (TTATG[TC]AA), CRE (TGACGTCA), Pdp1 core (ATTTAT/ATAAAT), and PER-repeat core (CATAC/GTATG), following motif definitions used in insect circadian studies [54,55]. Because short motifs are frequently palindromic or near-palindromic, they may function in either orientation; for reporting consistency we list positions on the + strand of the extracted promoter sequence but do not assume strand-restricted activity biologically.

Importantly, the presence of short cis-elements is only predictive of regulatory potential. Given their high background occurrence rate, motif presence alone does not demonstrate functional clock regulation: formal enrichment testing (for example, comparing motif counts or density in the *AmMTR* 3 kb promoter to counts from randomly sampled Amel_HAv3.1 promoters with appropriate multiple-testing correction) and orthogonal experimental validation (reporter assays, ChIP, or time-series expression and perturbation experiments) are required to establish functional circadian control.

### 4.8. Three-Dimensional Homology Modeling, Refinement, Docking and Validation of Apis mellifera MT2

#### 4.8.1. Structural Retrieval

All atoms 3D structural model was built utilizing the *A. mellifera* MT2 receptor (XP_392683.1) sequence with the aid of PHYRE 2.2 web server utilizing the Alpha Thread mode [71]. As AlphaThread performs template-based threading onto structurally related templates rather than ab initio deep-learning prediction, and because the highest-scoring templates exhibit low sequence identity to the target (see Results), local geometry—particularly flexible extracellular loops and side-chain conformations within the ligand-binding pocket—should be interpreted with caution. After model building the model was validated against the Ramchandran map then refined utilizing the Galaxy WEB server [72]. The best three models after refinement were utilized in the docking experiments to account for model-dependent variability. On the other hand, the structures of melatonin, 2-phenylmelatonin, and luzindole were retrieved from PubChem database [73].

#### 4.8.2. Sequence Alignment and Docking Calculations

Pairwise sequence alignment was made by Clustal omega webserver then visualized by ESPript 3.0 for *Homo sapiens* and *A. mellifera* MT2 receptors [74]. AutoDock Vina was utilized to dock melatonin, 2-phenylmelatonin, and luzindole into the *A. mellifera* MT2 receptor model at the suggested binding site defined by: R0110, V114, Y111, T165, F173, I184, K194, L197, F198, Y272, L273, Y295, L298, and Y299 residues [49]. These residues resemble the 2-phenylmelatonin binding pocket of human MT2 receptor (retrieved from PDB ID: 6ME6) M120, V124, I125, N175, L181, F192, Q194, Y200, V205, W264, L267, Y294, A297, and Y298 [47]. In the docking calculations the default parameters are used except that we increased the exhaustiveness to 64 to obtain more accurate results for the docked ligands. The search box was set to the size of 56 × 52 × 58 Å^3^ and entered at (3.9, 3.1, −10.8) Å with spacing of 0.375 Å. The protein-ligand interaction profiler (PLIP, version 3.0.0) was utilized to mine the docking complexes with the aid of PyMOL software (Schrödinger, New York, NY, USA; version 3.1.6.1) to visualize the complexes [75,76]. All docking calculations were performed across the three refined MT2 structural models, and the resulting docking energies and interaction counts are reported as the mean (±standard deviation) to reflect variability arising from model-dependent structural differences.

## 5. Conclusions

Our behavioral, pharmacological, and RNAi data demonstrate that melatonin signaling modulates behavioral maturation in *A. mellifera*, promoting metabolic preparedness (α-glucosidase trend) and outward activity (reduced hive retention, accelerated waggle-dance onset). Receptor perturbation shows that intact AmMTR expression is necessary for normal foraging propensity. Collectively these results position melatonin as an important, clock-linked neuroendocrine layer that complements—and likely interacts with—classic JH–Vg and biogenic amine pathways, rather than replacing them. Disentangling the mechanistic relationships among these axes and quantifying the temporal dynamics of endogenous melatonin should be priorities for future work given their potential relevance to colony resilience and the sublethal effects of agrochemicals.

## Figures and Tables

**Table 1:** The validation of the initial model predicted by PHYRE 2.2 alpha thread mode and the refined five models by Galaxy WEB server.

Model	RMSD (Å)	MolProbity	Clash Score	Poor Rotamers	Ramachandran Favored %
Initial	0.000	2.252	34.3	0.0	96.4
MODEL 1	0.240	1.382	6.9	0.4	98.6
MODEL 2	0.249	1.473	8.8	0.0	98.6
MODEL 3	0.250	1.393	7.2	0.0	98.6
MODEL 4	0.242	1.382	6.9	0.8	98.6
MODEL 5	0.232	1.482	9.0	0.0	98.6

Models: ‘Initial’ = PHYRE2 (Alpha Thread) output; MODEL 1–5 = GalaxyWEB-refined models. RMSD measured relative to the initial model. MolProbity, clash score, poor rotamers and Ramachandran statistics reported from MolProbity validation. Higher ‘Ramachandran favored %’ and lower clash/rotamer scores indicate improved stereochemical quality after refinement. Values derived from the refined models used for docking.

**Table 2:** Protein–ligand interaction summary for melatonin, 2-phenylmelatonin and luzindole docked into *Apis mellifera* MT2 (three refined models). Numbers indicate the count of interactions observed per model; residues list specific contacts identified by PLIP across models 1–3. Interactions were categorized as hydrogen bonds (H-bonds), hydrophobic contacts, and π-cation interactions; docking was performed with AutoDock Vina (exhaustiveness = 64) and interactions mined with PLIP.

Ligand	Protein Model	H-Bonds		Hydrophobic Contacts		π-Cation Interaction	
		Number	Residues	Number	Residues	Number	Residues
Melatonin	Model 1			1	L298		
	Model 2			3	R110, V114 and L298		
	Model 3			2	I275 and L298		
2-phenylmelatonin	Model 1			2	Y272 and L298	1	K194
	Model 2			3	Q162, I184, and L197	1	K194
	Model 3			2	Y272 and L273	1	K194
Luzindole	Model 1	1	K279	2	I275 and L298		
	Model 2			2	K194 and F198		
	Model 3	1	K279	1	L298	1	K279

Interaction counts reflect PLIP-annotated contacts in each model. Residues noted correspond to the search box/binding site region defined for docking (see Methods). Differences between models reflect refinement-dependent side-chain conformations; recurring residues (e.g., L298, K194, K279) denote conserved contact points across models and ligands. Comparative human MT2 binding residues (PDB 6ME6) are discussed in the text.

## Data Availability

All data generated or analyzed during this study are included in this article.

## Supplementary Materials

The following supporting information can be downloaded at: https://www.mdpi.com/article/10.3390/ijms27010035/s1.

## Abbreviations

The following abbreviations are used in this manuscript:

5-HT	Serotonin
AANAT	Arylalkylamine *N*-Acetyltransferase
AccMTNR1A	*Apis cerana cerana* Melatonin Receptor Type 1A
AmMTR/AmMT2	*Apis mellifera* Melatonin Receptor (Putative)
AmNAT1	*Apis mellifera* Arylalkylamine *N*-Acetyltransferase 1
AmNAT2	*Apis mellifera* Arylalkylamine *N*-Acetyltransferase 2
BR–SOG	Brain–Suboesophageal Ganglion
cDNA	Complementary DNA
CLES	Common-Language Effect Size
CRE	cAMP Response Element
CREB1	cAMP Response Element-Binding Protein 1
DBP	D-site Binding Protein
dH_2_O	Distilled Water
DNA	Deoxyribonucleic Acid
DNase	Deoxyribonuclease
dsRNA	Double-Stranded RNA
E-box	Enhancer Box (DNA motif)
FASTA	Fast-All (a sequence format)
FIMO	Find Individual Motif Occurrences
GFP	Green Fluorescent Protein
GPCR	G Protein-Coupled Receptor
HPGs	Hypopharyngeal Glands
IQR	Interquartile Range
JH	Juvenile Hormone
MT2	Melatonin Receptor Type 2
OA	Octopamine
PBS	Phosphate-Buffered Saline
PCR	Polymerase Chain Reaction
Pdp1	Par Domain Protein 1
PER	Period (circadian clock protein)
PLIP	Protein-Ligand Interaction Profiler
PTTH	Prothoracicotropic Hormone
PWM	Position Weight Matrix
qPCR/qRT-PCR	Quantitative (Real-Time) Polymerase Chain Reaction
RNA	Ribonucleic Acid
RNAi	RNA Interference
RNAiso Plus	A commercial reagent for RNA extraction
RPL32/rp49	Ribosomal Protein L32 (a common reference gene)
RMSD	Root-Mean-Square Deviation
SAR	Structure-Activity Relationship
SD	Standard Deviation
TSS	Transcription Start Site
T7	T7 Bacteriophage (promoter sequence)
UTR	Untranslated Region
Vg	Vitellogenin
*v*/*v*	Volume per Volume
*w*/*v*	Weight per Volume

## References

[1] Johnson B.R. (2010). Division of labor in honeybees: Form, function, and proximate mechanisms. Behav. Ecol. Sociobiol..

[2] Hamilton A.R., Traniello I.M., Ray A.M., Caldwell A.S., Wickline S.A., Robinson G.E. (2019). Division of labor in honey bees is associated with transcriptional regulatory plasticity in the brain. J. Exp. Biol..

[3] Zhang X., Hao Y., Niu Q., Chen Y., Xia Z., Xie Z., Zhao Y., Kong L., Peng W. (2022). Division of labor among worker bees is associated with the lipidomic plasticity in their brains. Agriculture.

[4] Schilcher F., Scheiner R. (2023). New insight into molecular mechanisms underlying division of labor in honeybees. Curr. Opin. Insect Sci..

[5] Lau P., Lesne P., Payne A.N., Garcia C., Gomez J., Behmer S.T., Rangel J. (2025). Do not compromise: Nurse honeybees practice strict protein-lipid regulation. iScience.

[6] Knecht D., Kaatz H.H. (1990). Patterns of larval food production by hypopharyngeal glands in adult worker honey bees. Apidologie.

[7] Akülkü İ., Ghanem S., Filiztekin E., Suwannapong G., Mayack C. (2021). Age-dependent honey bee appetite regulation is mediated by trehalose and octopamine baseline levels. Insects.

[8] Simpson J., Inge B.M., Wilding N. (1968). Invertase in hypopharyngeal glands of the honeybee. J. Apic. Res..

[9] Ohashi K., Natori S., Kubo T. (1999). Expression of amylase and glucose oxidase in the hypopharyngeal gland with an age-dependent role change of the worker honeybee (*Apis mellifera* L.). Eur. J. Biochem..

[10] Ohashi K., Sasaki M., Sasagawa H., Nakamura J., Natori S., Kubo T. (2000). Functional flexibility of the honey bee hypopharyngeal gland in a dequeened colony. Zool. Sci..

[11] Ohashi K., Sawata M., Takeuchi H., Natori S., Kubo T. (1996). Molecular cloning and expression of the gene for α-glucosidase, a nectar-processing enzyme, from forager honeybee *Apis mellifera*. Biochem. Biophys. Res. Commun..

[12] Kubota M., Tsuji M., Nishimoto M., Wongchawalit J., Okuyama M., Mori H., Matsui H., Surarit R., Svasti J., Kimura A. (2004). Localization of alpha-glucosidases I, II, and III in organs of European honeybees, *Apis mellifera* L., and the origin of alpha-glucosidase in honey. Biosci. Biotechnol. Biochem..

[13] Kaewmuangmoon J., Kilaso M., Leartsakulpanich U., Kimura K., Kimura A., Chanchao C. (2013). Expression of a secretory α-glucosidase II from *Apis cerana indica* in *Pichia pastoris* and its characterization. BMC Biotechnol..

[14] Fent K., Haltiner T., Kunz P., Christen V. (2020). Insecticides cause transcriptional alterations of endocrine related genes in the brain of honey bee foragers. Chemosphere.

[15] Christen V. (2023). Different effects of pesticides on transcripts of the endocrine regulation and energy metabolism in honeybee foragers from different colonies. Sci. Rep..

[16] Siehler O., Wang S., Bloch G. (2021). Social synchronization of circadian rhythms with a focus on honeybees. Philos. Trans. R. Soc. Lond. B Biol. Sci..

[17] Corona M., Branchiccela B., Alburaki M., Palmer-Young E.C., Madella S., Chen Y., Evans J.D. (2023). Decoupling the effects of nutrition, age, and behavioral caste on honey bee physiology, immunity, and colony health. Front. Physiol..

[18] Elekonich M., Schulz D.J., Bloch G., Robinson G.E. (2001). Juvenile hormone levels in honey bee (*Apis mellifera* L.) foragers: Foraging experience and diurnal variation. J. Insect Physiol..

[19] Bloch G., Sullivan J.P., Robinson G.E. (2002). Juvenile hormone and circadian locomotor activity in the honey bee *Apis mellifera*. J. Insect Physiol..

[20] Amdam G.V., Nilsen K.A., Norberg K., Fondrk M.K., Hartfelder K. (2007). Variation in endocrine signaling underlies variation in social life history. Am. Nat..

[21] Nelson C.M., Ihle K.E., Fondrk M.K., Page R.E., Amdam G.V. (2007). The gene vitellogenin has multiple coordinating effects on social organization. PLoS Biol..

[22] Sullivan J.P., Fahrbach S.E., Robinson G.E. (2000). Juvenile hormone paces behavioral development in the adult worker honey bee. Horm. Behav..

[23] Harris J.W., Woodring J. (1992). Effects of stress, age, season, and source colony on levels of octopamine, dopamine and serotonin in the honey bee (*Apis mellifera* L.) brain. J. Insect Physiol..

[24] Chen Y.L., Hung Y.S., Yang E.C. (2008). Biogenic amine levels change in the brains of stressed honeybees. Arch. Insect Biochem. Physiol..

[25] Beggs K.T., Mercer A.R. (2009). Dopamine receptor activation by honey bee queen pheromone. Curr. Biol..

[26] Wang Q., Mohamed A.A.M., Takeda M. (2013). Serotonin receptor B may lock the gate of PTTH release/synthesis in the Chinese silk moth, *Antheraea pernyi*: A diapause initiation/maintenance mechanism?. PLoS ONE.

[27] Raza M.F., Li W. (2025). Biogenic amines in honey bee cognition: Neurochemical pathways and stress impacts. Curr. Opin. Insect Sci..

[28] Bloch G., Meshi A. (2007). Influences of octopamine and juvenile hormone on locomotor behavior and period gene expression in the honeybee, *Apis mellifera*. J. Comp. Physiol. A Neuroethol. Sens. Neural Behav. Physiol..

[29] Moore D., Angel J.E., Cheeseman I.M., Fahrbach S.E., Robinson G.E. (1998). Timekeeping in the honey bee colony: Integration of circadian rhythms and division of labor. Behav. Ecol. Sociobiol..

[30] Bloch G., Solomon S.M., Robinson G.E., Fahrbach S.E. (2003). Patterns of PERIOD and pigment-dispersing hormone immunoreactivity in the brain of the European honeybee (*Apis mellifera*): Age- and time-related plasticity. J. Comp. Neurol..

[31] Bloch G., Rubinstein C.D., Robinson G.E. (2004). period expression in the honey bee brain is developmentally regulated and not affected by light, flight experience, or colony type. Insect Biochem. Mol. Biol..

[32] Beer K., Helfrich-Förster C. (2020). Post-embryonic development of the circadian clock seems to correlate with social lifestyle in bees. Front. Cell Dev. Biol..

[33] Araujo N.S., Arias M.C. (2021). Gene expression and epigenetics reveal species-specific mechanisms acting upon common molecular pathways in the evolution of task division in bees. Sci. Rep..

[34] Izawa N., Hiragaki S., Mohamed A.A., Elgendy A.M., Ohtani T., Takeda M. (2023). Arylalkylamine *N*-acetyltransferase activity parallel to work types and their temporal shift suggests its involvement in polyethism regulation in *Apis mellifera* workers. Apidologie.

[35] Ganguly S., Klein D.C., Kumar V. (2017). The timezyme and melatonin: Essential elements of vertebrate timekeeping. Biological Timekeeping: Clocks, Rhythms and Behaviour.

[36] O’Flynn B.G., Suárez G., Hawley A.J., Merkler D.J. (2018). Insect arylalkylamine *N*-acetyltransferases: Mechanism and role in fatty acid amide biosynthesis. Front. Mol. Biosci..

[37] Kim T.K., Atigadda V.R., Brzeminski P., Fabisiak A., Tang E.K.Y., Tuckey R.C., Reiter R.J., Slominski A.T. (2021). Detection of serotonin, melatonin, and their metabolites in honey. ACS Food Sci. Technol..

[38] Yang L., Qin Y., Li X., Song D., Qi M. (2007). Brain melatonin content and polyethism in adult workers of *Apis mellifera* and *Apis cerana* (Hym., Apidae). J. Appl. Entomol..

[39] Mohamed A.A.M., Wang Q., Bembenek J., Ichihara N., Hiragaki S., Suzuki T., Takeda M. (2014). N-acetyltransferase (nat) is a critical conjunct of photoperiodism between the circadian system and endocrine axis in *Antheraea pernyi*. PLoS ONE.

[40] Huang Z., Lin R., Yu R. Why nurse bees do not sleep: Melatonin implicated. Proceedings of the ESA 2001 Annual Meeting—2001: An Entomological Odyssey of ESA.

[41] Fan W., Li G., Zhang X., Wang Y., Wang C., Xu B., Guo X., Li H. (2021). The role of melatonin and tryptophan-5-hydroxylase-1 in different abiotic stressors in *Apis cerana cerana*. J. Insect Physiol..

[42] Li Z., Duan J., Chen L., Wang Y., Qin Q., Dang X., Zhou Z. (2022). Melatonin enhances the antioxidant capacity to rescue the honey bee *Apis mellifera* from the ecotoxicological effects caused by environmental imidacloprid. Ecotoxicol. Environ. Saf..

[43] Li G., Zhang Y., Ni Y., Wang Y., Xu B., Guo X. (2018). Identification of a melatonin receptor type 1A gene (AccMTNR1A) in *Apis cerana cerana* and its possible involvement in the response to low temperature stress. Sci. Nat..

[44] Richter K., Peschke E., Peschke D. (2000). A neuroendocrine releasing effect of melatonin in the brain of an insect, *Periplaneta americana* (L.). J. Pineal Res..

[45] Richter K., Pesche E., Pesche D. (1999). Effect of melatonin on the release of prothoracicotropic hormone from the brain of *Periplaneta americana* (Blattodea: Blattidae). Eur. J. Entomol..

[46] Kamruzzaman A.S.M., Hiragaki S., Watari Y., Natsukawa T., Yasuhara A., Ichihara N., Mohamed A.A., Elgendy A.M., Takeda M. (2021). Clock-controlled arylalkylamine *N*-acetyltransferase (aaNAT) regulates circadian rhythms of locomotor activity in the American cockroach, *Periplaneta americana*, via melatonin/MT2-like receptor. J. Pineal Res..

[47] Johansson L.C., Stauch B., McCorvy J.D., Han G.W., Patel N., Huang X.P., Batyuk A., Gati C., Slocum S.T., Li C. (2019). XFEL Structures of the human MT2 melatonin receptor reveal the basis of subtype selectivity. Nature.

[48] Stauch B., Johansson L.C., Cherezov V. (2020). Structural insights into melatonin receptors. FEBS J..

[49] Eberhardt J., Santos-Martins D., Tillack A.F., Forli S. (2021). AutoDock Vina 1.2.0: New docking methods, expanded force field, and python bindings. J. Chem. Inf. Model..

[50] Giray T., Galindo-Cardona A., Oskay D. (2007). Octopamine influences honey bee foraging preference. J. Insect Physiol..

[51] Giannoni-Guzmán M.A., Rivera-Rodriguez E.J., Aleman-Rios J., Melendez Moreno A.M., Pérez Ramos M., Pérez-Claudio E., Loubriel D., Moore D., Giray T., Agosto-Rivera J.L. (2021). The role of colony temperature in the entrainment of circadian rhythms of honey bee foragers. Ann. Entomol. Soc. Am..

[52] Toma D.P., Bloch G., Moore D., Robinson G.E. (2000). Changes in period mRNA levels in the brain and division of labor in honey bee colonies. Proc. Natl. Acad. Sci. USA.

[53] Zhou Q., Yang D., Wu M., Guo Y., Guo W., Zhong L., Cai X., Dai A., Jang W., Shakhnovich E.I. (2019). Common activation mechanism of class A GPCRs. eLife.

[54] Cyran S.A., Buchsbaum A.M., Reddy K.L., Lin M.C., Glossop N.R.J., Hardin P.E., Young M.W., Storti R.V., Blau J. (2003). vrille, Pdp1, and dClock form a second feedback loop in the *Drosophila* circadian clock. Cell.

[55] Hardin P.E. (2011). Molecular genetic analysis of circadian timekeeping in *Drosophila*. Adv. Genet..

[56] Huang J., Zhang Z., Feng W., Zhao Y., Aldanondo A., de Brito Sanchez M.G., Paoli M., Rolland A., Li Z., Nie H. (2022). Food wanting is mediated by transient activation of dopaminergic signaling in the honey bee brain. Science.

[57] Zhao D., Yu Y., Shen Y., Liu Q., Zhao Z., Sharma R., Reiter R.J. (2019). Melatonin synthesis and function: Evolutionary history in animals and plants. Front. Endocrinol..

[58] Song Y., Yoon M. (2025). Melatonin effects on animal behavior: Circadian rhythm, stress response, and modulation of behavioral patterns. J. Anim. Sci. Technol..

[59] McHenry L.C., Schürch R., Council-Troche M., Gross A.D., Johnson L.E., Ohlinger B.D., Couvillon M.J. (2025). Sublethal glyphosate exposure reduces honey bee foraging and alters the balance of biogenic amines in the brain. J. Exp. Biol..

[60] Deeter M.E., Snyder L.A., Meador C., Corby-Harris V. (2023). Accelerated abdominal lipid depletion from pesticide treatment alters honey bee pollen foraging strategy but not onset. J. Exp. Biol..

[61] Hiragaki S., Suzuki T., Mohamed A.A., Takeda M. (2015). Structures and functions of insect arylalkylamine *N*-acetyltransferase (iaaNAT); a key enzyme for physiological and behavioral switch in arthropods. Front. Physiol..

[62] Koto A., Tamura M., Wong P.S., Aburatani S., Privman E., Stoffel C., Crespi A., McKenzie S.K., La Mendola C., Kay T. (2023). Social isolation shortens lifespan through oxidative stress in ants. Nat. Commun..

[63] Shemesh Y., Eban-Rothschild A., Cohen M., Bloch G. (2010). Molecular dynamics and social regulation of context-dependent plasticity in the circadian clockwork of the honey bee. J. Neurosci..

[64] Fuchikawa T., Shimizu I. (2007). Effects of temperature on circadian rhythm in the Japanese honeybee, *Apis cerana japonica*. J. Insect Physiol..

[65] Lourenço A.P., Mackert A., dos Santos Cristino A., Simões Z.L.P. (2008). Validation of reference genes for gene expression studies in the honey bee, *Apis mellifera*, by quantitative real-time RT-PCR. Apidologie.

[66] Dussaubat C., Brunet J.-L., Higes M., Colbourne J.K., Lopez J., Choi J.-H., Martín-Hernández R., Botías C., Cousin M., McDonnell C. (2012). Gut pathology and responses to the microsporidium *Nosema ceranae* in the honey bee *Apis mellifera*. PLoS ONE.

[67] Livak K.J., Schmittgen T.D. (2001). Analysis of relative gene expression data using real-time quantitative PCR and the 2^−ΔΔCT^ method. Methods.

[68] R Core Team (2022). R: A Language and Environment for Statistical Computing.

[69] Grant C.E., Bailey T.L., Noble W.S. (2011). FIMO: Scanning for occurrences of a given motif. Bioinformatics.

[70] Castro-Mondragon J.A., Riudavets-Puig R., Rauluseviciute I., Lemma R.B., Turchi L., Blanc-Mathieu R., Lucas J., Boddie P., Khan A., Manosalva Pérez N. (2022). JASPAR 2022: The 9th release of the open-access database of transcription factor binding profiles. Nucleic Acids Res..

[71] Powell H.R., Islam S.A., David A., Sternberg M.J.E. (2025). Phyre2.2: A community resource for template-based protein structure prediction. J. Mol. Biol..

[72] Seok C., Baek M., Steinegger M., Park H., Lee G.R., Won J. (2021). Accurate protein structure prediction: What comes next?. BioDesign.

[73] Kim S., Chen J., Cheng T., Gindulyte A., He J., He S., Li Q., Shoemaker B.A., Thiessen P.A., Yu B. (2025). PubChem 2025 update. Nucleic Acids Res..

[74] Robert X., Gouet P. (2014). Deciphering key features in protein structures with the new ENDscript server. Nucleic Acids Res..

[75] Yuan S., Chan H.C.S., Hu Z. (2017). Using PyMOL as a platform for computational drug design. Wiley Interdiscip. Rev. Comput. Mol. Sci..

[76] Schake P., Bolz S.N., Linnemann K., Schroeder M. (2025). PLIP 2025: Introducing protein–protein interactions to the protein–ligand interaction profiler. Nucleic Acids Res..

