# Supplementary material for: Melatonergic Regulation of Polyethism and Circadian Foraging in Apis mellifera
Source: Int J Mol Sci. 2025 Dec 19;27(1):35. doi: 10.3390/ijms27010035 (PMC12786324; doi:10.3390/ijms27010035)
Supplement: Supplementary file 1 [file ijms-27-00035-s001.zip › ijms-4005473-supplementary.pdf]

Table S1. Predicted circadian cis-regulatory motifs in the *Apis mellifera MTR* promoter. Motifs were identified within the 3 kb upstream region of the *MTR* transcription start site (TSS; NC_037647.1:5,949,179, Amel_HAv3.1 assembly) using two complementary approaches: (i) position weight matrix (PWM; FIMO-style) scanning, and (ii) consensus/regex search for canonical or degenerate sequences. A total of 23 sequence-verified motifs were retained, comprising 11 PWM-derived high-confidence matches and 12 consensus/regex matches. Coordinates are expressed relative to the annotated TSS (negative = upstream). All reported sequences were verified against the genomic FASTA. Multiple Pdp1 core elements cluster proximal to the TSS (−820, −357, −102 bp), suggesting potential regulatory importance.

Consensus motif	Position (bp, rel. to TSS)	Strand	Match sequence	Detection method
E-box (CANNTG)	−2762	+	CATTTG	Regex
Pdp1 core	−2739	+	ATAAAT	Regex
DBP	−2701	+	TGTGATATAATA	PWM
DBP	−2498	+	TATTACAAAATA	PWM
Pdp1 core	−2140	+	ATAAAT	Regex
DBP	−1987	+	AATTACGTAAAA	PWM
DBP	−1860	+	AATCACGCAATC	PWM
Pdp1 core	−1798	+	ATTTAT	Regex
PERR core	−1673	+	CATAC	Regex
E-box (CANNTG)	−1580	+	CAGTTG	Regex
PERR core	−1400	+	GTATG	Regex
E-box (CANNTG)	−1383	+	CAAGTG	Regex
CLOCK	−1334	+	TATACGTGTT	PWM
DBP	−1185	+	TATTATATTATT	PWM
Pdp1 core	−1229	+	ATAAAT	Regex
Pdp1 core	−1193	+	ATAAAT	Regex
Pdp1 core	−820	+	ATTTAT	Regex
DBP	−457	+	TATTTTATAATA	PWM
DBP	−417	+	AGTTGCATCATT	PWM
Pdp1 core	−357	+	ATTTAT	Regex
DBP	−298	+	TATTCTGTAATA	PWM
DBP	−132	+	GATTACATTACA	PWM
Pdp1 core	−102	+	ATTTAT	Regex
CREB1 (CRE)	−93	+	TGTCGTCA	PWM

Consensus motifs included canonical E-box (CACGTG), degenerate E-box (CANNTG), D-box (TTATG[TC]AA), CRE (TGACGTCA), Pdp1 core (ATTTAT/ATAAAT), and PER-repeat core (CATAC/GTATG).

Table S2. Primer sequences used in this study for RT-PCR, qPCR, and dsRNA synthesis in *Apis mellifera*.

Target gene	Accession no.	Primer name	Sequence (5′→3′)	Source / Reference
Melatonin receptor (*AmMTR*, LOC409159)	XM_392683.6	MelR_RT-PCR_F	ATCGTAAGAGATAGCAGAGG	This study
		MelR_RT-PCR_R	ATGGTGATTGGCAGGTAA	
		MelR_qPCR_F	TGTAGCAGACTTCGTGTT	This study
		MelR_qPCR_R	AATATACCTGTTGATAGTGATAGC	This study
		MelR_dsRNA_F_T7	TAATACGACTCACTATAGGGGGGAACCCTGTTAGGAATTTGGGG	This study
		MelR_dsRNA_R_T7	TAATACGACTCACTATAGGGGGGATGGTGATTGGCAGGTAACAA	This study
GFP (control)	–	GFP-T7-F	TAATACGACTCACTATAGGGGAGACCTGAAGTTCATCTGCACCAC	[39]
		GFP-T7-R	TAATACGACTCACTATAGGGGAGAACGAACTCCAGCAGGACCAT	[39]
AANAT1	XM_026443226.1	AANAT1_qF	CATCAATCGGTACAGCTCTC	This study
		AANAT1_qR	ACGTCCTCGACAATACGTGA	This study
AANAT2	XM_026443227.1	AANAT2_qF	ACCCTCCCTATTTGCTCCAACT	This study
		AANAT2_qR	ACGACTTTTCGTGCGTTTTCG	This study
α-*Glucosidase* (*Hbg3*)	NM_001040236	Hbg3-F	TTGCTGCCAGGTGTTGCCGT	[66]
		Hbg3-R	TTGGAATGGCGTTCTCGCGGG	[66]
Actin	XM_623378	Actin-F	ACCAGAGAGGAAGTACTCTG	[39]
		Actin-R	AGATTCACGTGGACATTTCAGC	[39]
rp49 (*RPL32*)	DQ296005.1	RP49-F	CGTCATATGTTGCCAACTGGT	[65]
		RP49-R	TTGAGCACGTTCAACAATGG	[65]

Underlined sequences are the T7 promoter. Amplicon size (bp; target region, T7 excluded). Primers were designed using Primer3 and checked for specificity against the *Apis mellifera* RefSeq using Primer-BLAST; qPCR amplicon sizes refer to the target region only (T7 promoter sequences appended to dsRNA primers are excluded).
